# Chronic insomnia remitting after maxillomandibular advancement for mild obstructive sleep apnea: a case series

**DOI:** 10.1186/s13256-019-2182-9

**Published:** 2019-08-14

**Authors:** Michael Proothi, Victor J. R. Grazina, Avram R. Gold

**Affiliations:** 1 Stony Brook University Sleep Disorders Center, Renaissance School of Medicine at Stony Brook University, 240 Middle Country Road, Smithtown, NY 11787 USA; 2 Department of Oral and Maxillofacial Surgery, Stony Brook University School of Dental Medicine, Stony Brook, NY 11794-8705 USA; 3 Stony Brook Oral and Facial Surgery, 207 Hallock Road #2, Stony Brook, NY 11790 USA; 4 Bach and Grazina Orthodontics, 235 Osborn Avenue #2, Riverhead, NY 11901 USA; 5 Department of Medicine, Renaissance School of Medicine at Stony Brook University, 101 Nicolls Road, Stony Brook, NY 11794 USA

**Keywords:** Insomnia, Stress, Somatic arousal, Bipolar disorder, Temporomandibular joint syndrome, Obstructive sleep apnea, Maxillomandibular advancement

## Abstract

**Background:**

Chronic insomnia and obstructive sleep apnea are both common sleep disorders. Chronic insomnia is thought to result from stress-related physiologic hyperarousal (*somatic arousal*) that makes it difficult for an individual to fall or stay asleep. Obstructive sleep apnea is thought to result from obstructive respiratory events causing arousals, sleep fragmentation, and recurrent oxygen desaturation. Although the two disorders seem different, they predispose to the same long-term, stress-related illnesses, and when they occur in the same individual, each affects the other’s response to treatment; they interact. This report of three cases describes patients with both chronic insomnia and obstructive sleep apnea in whom the chronic insomnia remitted with no specific treatment following treatment of obstructive sleep apnea with maxillomandibular advancement.

**Case presentations:**

Our three Caucasians patients each presented with severe, chronic insomnia associated with somatic arousal and fatigue occurring either alone, in association with bipolar disorder, or with temporomandibular joint syndrome. Polysomnography revealed that each patient also had mild obstructive sleep apnea, despite only one snoring audibly. One patient experienced a modest improvement in her somatic arousal, insomnia severity, and fatigue with autotitrating nasal continuous positive airway pressure, but the other two did not tolerate nasal continuous positive airway pressure. None of the patients received treatment for insomnia. All three patients subsequently underwent maxillomandibular advancement to treat mild obstructive sleep apnea and experienced prolonged, complete resolution of somatic arousal, chronic insomnia, and fatigue. The patient with bipolar disorder also experienced complete remission of his symptoms of depression during the 1 year he was followed postoperatively.

**Conclusions:**

These three cases lend support to the hypothesis that chronic insomnia and obstructive sleep apnea share a pathophysiology of chronic stress. Among patients with obstructive sleep apnea, the stress response is directed at inspiratory airflow limitation during sleep (hypopnea, snoring, and inaudible fluttering of the throat). Therefore, when chronic insomnia and obstructive sleep apnea occur in one individual, aggressive treatment of obstructive sleep apnea may lead to a reduction in chronic stress that causes the patient’s chronic insomnia to remit.

## Background

Chronic insomnia is a state of delayed sleep onset and poor sleep maintenance lasting more than 6 months. It is widely hypothesized to be a disorder of physiologic hyperarousal [1] (also termed *somatic arousal*, the term we use throughout this report), a state of chronic stress characterized by increased sympathetic nervous system (SNS) activity [1]. The prevalence of chronic insomnia varies with the source, but it may affect 1 in 3 adults [2]. Because stress is believed to have a pathophysiologic role in chronic insomnia, it is not surprising that patients with chronic insomnia experience the health consequences of chronic stress, including hypersomnolence and fatigue, hypertension and cardiovascular disease, impaired glucose tolerance and diabetes, and anxiety and depression [3].

Obstructive sleep apnea (OSA) is recognized to be a disorder of inspiratory fluttering (hypopnea, snoring, and silent inspiratory airflow limitation) or collapse with complete occlusion of the pharyngeal airway (obstructive apnea) during sleep. It is widely hypothesized to be a disorder of sleep fragmentation by recurrent arousals and oxygen desaturation resulting from obstructed breathing [4] (known as the *sleep fragmentation paradigm* of OSA). Its prevalence, like that of chronic insomnia, varies with the source, but it may also be as high as 1 in 3 adults [5]. Although the pathophysiology of OSA is believed to differ from that of chronic insomnia, they share the health consequences of hypersomnolence and fatigue, hypertension and cardiovascular disease, impaired glucose tolerance and diabetes, and anxiety and depression [6–8]. The reasons for chronic insomnia and OSA, disparate sleep disorders, sharing the same health consequences remain unclear.

Sleep medicine professionals have long recognized that chronic insomnia and OSA often occur in the same individual [4, 9, 10]. Under such circumstances, the two conditions appear to interact. Patients with OSA with chronic insomnia seem not to tolerate treatment with nasal continuous positive airway pressure (CPAP) as well as do those without chronic insomnia [9, 10]. Patients with chronic insomnia with OSA appear to respond better to cognitive behavioral therapy if their OSA is first treated surgically (with pharyngoplasty and nasal surgery, as needed) [11]. Why chronic insomnia and OSA interact when occurring in the same individual remains unclear.

At the Stony Brook University Sleep Disorders Center (SBUSDC), our group views OSA as a disorder of *chronic stress* in which the brain’s limbic system responds to inspiratory airflow limitation during sleep (even mild snoring and silent inspiratory airflow limitation) with a stress response [12]. In support of our hypothesis, we have data from more than a decade of stress measurements in our patients. We measure stress in our patients using their self-reported frequency of symptoms of increased SNS activity, representing their self-reported somatic arousal level. Our instrument for this measurement, the Body Sensations Questionnaire (BSQ), measures the frequency of each of 17 symptoms using a 1 (absent) to 5 (very frequent) scale with increasing somatic arousal indicated by BSQ scores of 17 to 85. Our research has demonstrated that hypersomnolent patients with OSA have elevated BSQ scores compared with healthy control subjects [13] and that the sleepiness and fatigue levels of patients with OSA are positively correlated with their BSQ scores, representing their somatic arousal levels [14]. Moreover, nasal CPAP treatment of patients with OSA decreases not only their sleepiness and fatigue but also their somatic arousal as measured by the BSQ score [14].

The *chronic stress* paradigm of OSA suggests that when chronic insomnia and OSA occur in the same patient, it is the chronic stress of OSA that leads to the chronic symptom of insomnia. The BSQ data we have obtained from patients with OSA support this possibility, with lower BSQ scores (lower levels of stress) characterizing patients with OSA with sleep maintenance insomnia and higher BSQ scores characterizing those with both sleep onset and sleep maintenance insomnia [15]. If the chronic stress of OSA causes sleep onset and sleep maintenance insomnia when the two disorders coexist, then surgical cure of OSA might be expected to decrease somatic arousal and relieve the associated chronic insomnia in patients with both disorders. To further support the hypothesized relationship between the chronic stress of OSA and chronic insomnia, we present cases of three patients at the SBUSDC with both disorders who underwent maxillomandibular advancement (MMA) as treatment of their OSA. To describe their outcomes, we use not only the narrative from the medical record but also self-report questionnaire assessments of their insomnia (Insomnia Severity Index [ISI] [16]), their somatic arousal (BSQ [14, 15]), their fatigue (Fatigue Severity Scale [FSS] [17]), and their sleepiness (Epworth Sleepiness Scale [ESS] [18]) throughout their clinical evaluations, treatments, and follow-up.

## Case presentations

### Patient 1

Patient 1 was a 32-year-old Caucasian woman with a history of severe sleep onset and sleep maintenance insomnia of many years’ duration associated with severe daytime fatigue. The patient’s average bedtime was 11:00 p.m. She did not take hypnotics, and it took her 1–3 hours to fall asleep. About half the week, she experienced intrusive thoughts associated with her inability to fall asleep. She never experienced restless legs syndrome.

Once the patient was asleep, her bed partner did not witness snoring, episodes of apnea, or bruxism. She did not toss and turn during the night, and her bed was not in disarray in the morning. She experienced some sleep talking but no sleepwalking or sleep eating. She awakened several times per night, beginning a half-hour after falling sleep, choking and gasping as if she were strangling. After catching her breath, it took her anywhere from a half-hour to 2 hours to return to sleep. She experienced nightmares two or three nights per week.

The patient awakened to an alarm at 9 a.m. after five to nine hours of sleep. She was sometimes refreshed, and more sleep was more refreshing. She often awakened with a headache, dry mouth, nasal congestion, and a sore throat. The patient did not nap during the day, but she felt very sleepy while driving and sleepy/fatigued during her coursework as a graduate student in a health-related field.

The patient’s review of systems was remarkable for joint/muscle pain and feelings of “numbness and tingling.” She did not experience anxiety or depression. Her medical history was otherwise unremarkable, and her only medication was an oral contraceptive.

**Fig. 1:**
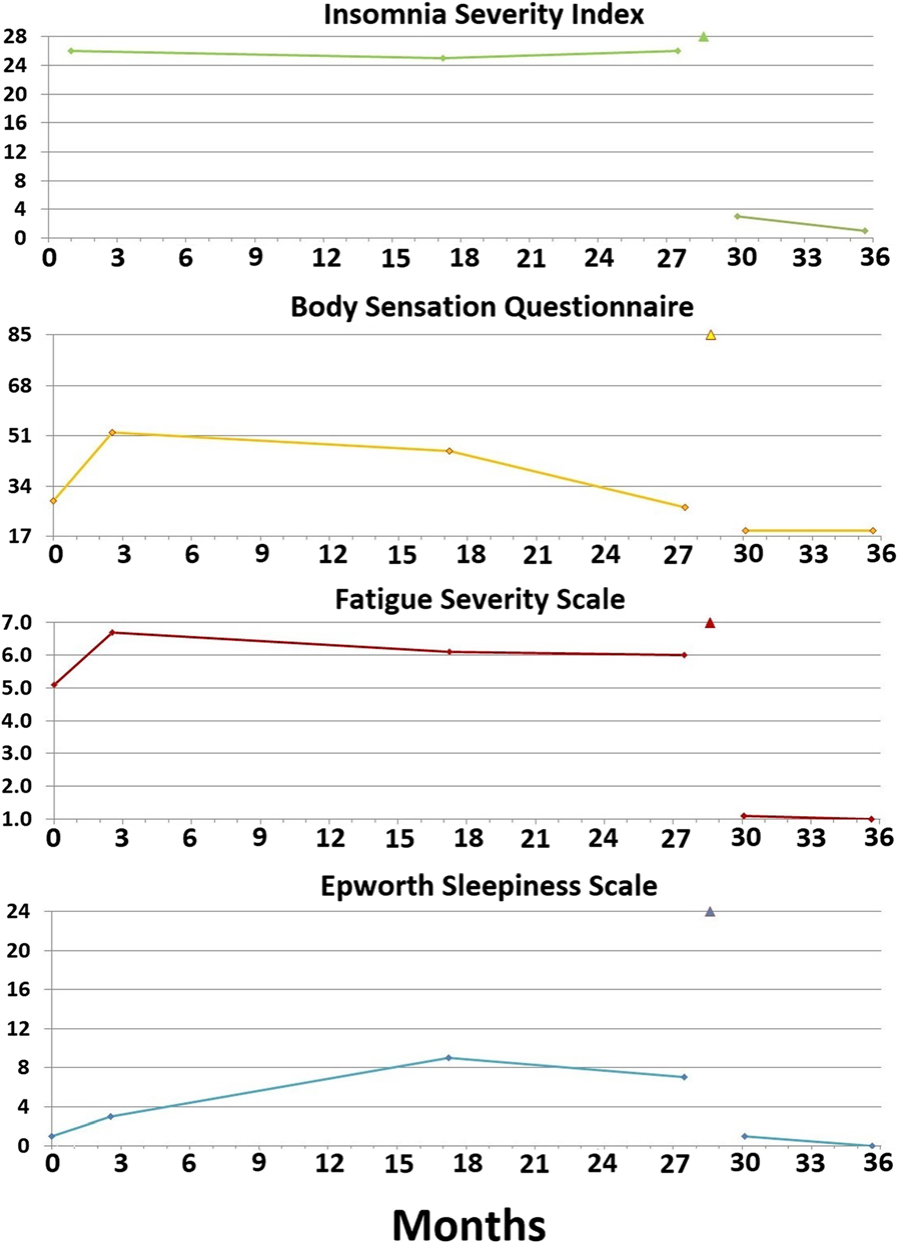
The scores of the four questionnaires completed by patient 1 to quantify the severity of her chronic insomnia (Insomnia Severity Index), somatic arousal (Body Sensations Questionnaire), fatigue (Fatigue Severity Scale), and sleepiness (Epworth Sleepiness Scale). The *x*-axis of each graph depicts the duration, in months, of our interaction with the patient, and the *y*-axis shows the entire score range for each questionnaire. The date of the surgical procedure is indicated by a marker on the top gridline of each graph. The figure indicates a high level of insomnia severity, fatigue severity, and somatic arousal for over 2 years prior to maxillomandibular advancement with a prompt decrease in the severity of all three symptoms postoperatively

Figure 1 illustrates the patient’s questionnaire scores at the time of her initial consultation. The patient’s ESS score was 1/24, indicating no sleepiness, and her FSS score was 5.1/7.0 (moderate to severe fatigue). Her ISI score was 26/28, indicating severe insomnia, and her BSQ score was 29/85, indicating the presence of somatic arousal. (In our previous research, healthy control subjects have scored at or below 20/85 [13, 19].)

**Fig. 2:**
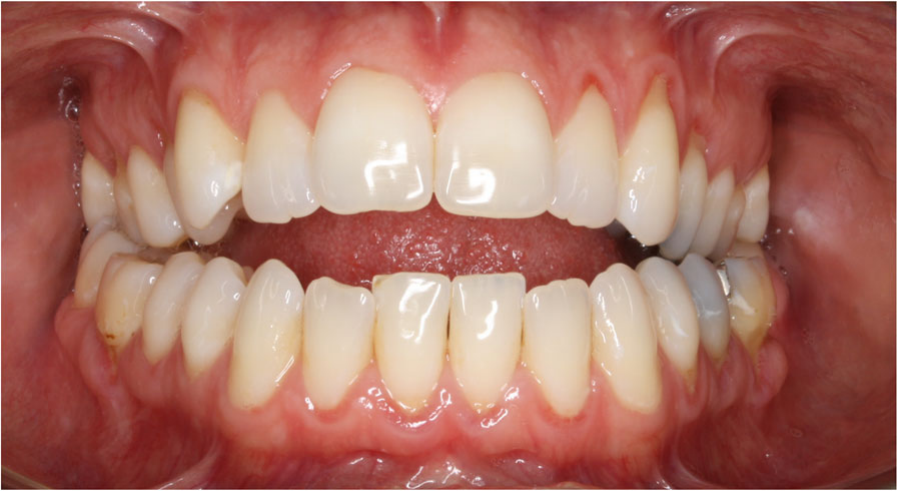
Patient 1’s “open bite.” With her molars opposed, the patient’s bite is open anteriorly with the tongue tip resting in the opening

On physical examination, the patient was 168 cm in height and weighed 62 kg (body mass index [BMI] 22.0 kg/m^2^). Her blood pressure was 134/87 mmHg, and her pulse was 75/minute. The patient’s appearance was that of midface (maxillary) hypoplasia with a protruding jaw. Examination of her mouth demonstrated an open bite (with her molars opposed, her bite remained open anteriorly; Fig. 2). Her hard palate was high-arched and narrow with an elongated soft palate. Her tongue appeared very large for the size of her oral cavity, with its tip resting in the open bite (Fig. 2). Her tonsils were not enlarged, and her Mallampati score was 2. The remainder of her examination was unremarkable.

The patient’s polysomnogram demonstrated a sleep latency of 189.5 minutes with a total sleep time of 138 minutes. Her sleep was divided into two approximately 1-hour periods composed largely of stages N1 and N2 with a small amount of N3 in each period. (Non-rapid eye movement [NREM] sleep is classified as three stages of increasing depth: N1, N2, and N3.) She had no rapid eye movement (REM) sleep. Her airflow pattern during sleep was largely mild inspiratory airflow limitation in the absence of audible snoring. Her apnea-hypopnea index was 0.4/hour, and her respiratory disturbance index (RDI) (including respiratory effort-related arousals) was 15.2/hour, consistent with mild to moderate OSA [20].

The patient returned to the sleep laboratory 1 week later for a nasal CPAP titration study, but she slept as poorly as during the diagnostic polysomnogram. Nasal CPAP at 6 cmH_2_O was prescribed, empirically, for the patient’s mild inspiratory airflow limitation. The patient attempted to sleep at home with nasal CPAP, but she was unable to tolerate it. Because of the patient’s young age, the severity of her insomnia and fatigue, her open bite, and the cosmetic implications of her maxillary hypoplasia, the sleep medicine physicians recommended MMA, and the patient agreed to a maxillofacial surgical consultation.

**Fig. 3:**
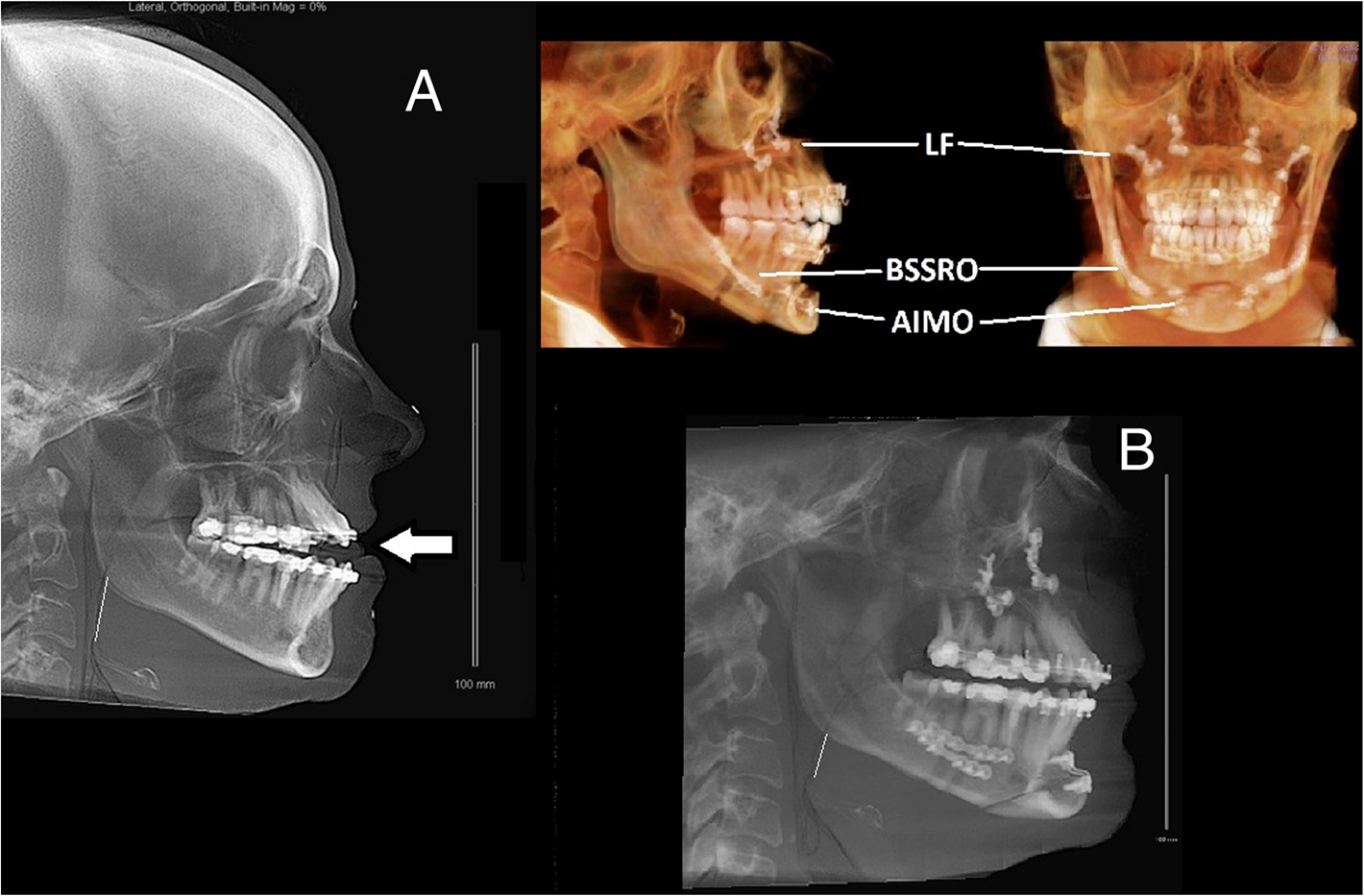
Lateral cephalograms of patient 1 preoperatively (**a**) and postoperatively (**b**), as well as an illustration of the surgery associated with the patient’s maxillomandibular advancement. **a** The patient’s midface hypoplasia, with the *horizontal arrow* highlighting the tip of the tongue in the open bite. The somewhat vertical white line placed over the base of the tongue illustrates its proximity to the posterior pharyngeal wall. The three elements of the maxillomandibular advancement are the LeFort 1 (LF), advancing the maxillae; the bilateral sagittal split ramus osteotomy (BSSRO), creating a “trombone slide” advancement of the mandible; and an anterior inferior mandibular osteotomy (AIMO) or “genioplasty,” moving forward the insertion of the genioglossus onto the mandible. **b** Postoperative lateral cephalogram of patient 1. The midface hypoplasia has been corrected, and the open bite has been closed by the orthodontic intervention. The vertical white line highlights the increased space between the base of the tongue and the posterior pharyngeal wall

The maxillofacial surgeon determined that the patient’s maxillae were retropositioned with a narrow nasal airway. Her mandible was not retropositioned, but it had to be advanced to widen her posterior airway space (Fig. 3a, left lateral cephalograph). The maxillofacial surgeon recommended a LeFort 1 maxillary advancement with separate advancement of each maxilla, positioning them to widen the nasal airway (Fig. 3); a bilateral sagittal split ramus osteotomy to elongate the body of the mandible, positioning the tongue forward (Fig. 3); and an anterior inferior mandibular osteotomy to further advance the tongue and create a more prominent chin (genioplasty) (Fig. 3). Before surgery, however, the patient underwent correction of her open bite by an orthodontist, a process that took approximately 1 year.

The patient underwent an MMA procedure 2 years and 4 months after her first sleep consultation (Fig. 3b, a postoperative left lateral cephalograph illustrating the orthodontics and MMA). On the first postoperative day, the patient had little swelling and reported to the surgeon that she had slept “very well” the preceding night. At 6 weeks, the patient reported it took 5 to 15 minutes for her to fall asleep, that she no longer woke up gasping, and that she returned to sleep quickly after awakenings, which were usually related to outside disturbances (her dog). She no longer experienced nightmares, and she no longer had joint/muscle pains, which she realized had been caused by “muscle tension.” The patient was no longer using any opioids and was using little analgesic of any kind for postoperative pain. The patient’s insomnia severity, level of somatic arousal, and fatigue severity had all remained consistently elevated for over 2 years until surgery was performed. All of her questionnaire scores then normalized within 6 weeks with no further intervention (Fig. 1). A few months postoperatively, the patient accepted a job offer in another state and did not return for a polysomnogram to objectively evaluate the effect of MMA on her sleep and breathing.

### Patient 2

Patient 2 was a 22-year-old Caucasian man referred by his psychiatrist for trouble with his sleep of many years’ duration. Sometimes, he slept throughout the night without issues but awakened unrefreshed and fatigued, unable to handle daily activities. Sometimes, he was unable to sleep for three or four consecutive nights and could not function at all. His inability to sleep was episodic, having no pattern and no trigger. He reported minimal caffeine intake and denied substance abuse or recreational drug use. He had tried a variety of medications, and none had improved his sleep (zolpidem, escitalopram, trazodone, and melatonin). He was unable to maintain a job or attend college, which was the reason he requested a sleep evaluation.

On nights that he could not fall asleep, the patient experienced intrusive thoughts but no restless legs syndrome. Once asleep, the patient did not snore, have witnessed apnea, or awaken choking or gasping. He did grind his teeth. He was a restless sleeper, and his bed was in disarray in the morning. He was not aware of walking or talking in his sleep. He experienced nightmares twice monthly.

During the patient’s childhood, his pediatrician had sent him for a polysomnogram. The result was “inconclusive,” and he never returned to the sleep medicine specialist for follow-up. At the age of 15, he had a palate expander for about 8 months, but he had “a lot of issues” with it, and it was discontinued. In addition to his insomnia, he had chronic sinus infections, anxiety, bipolar disorder, attention-deficit/hyperactivity disorder, migraine headaches, gastroesophageal reflux disease, and irritable bowel syndrome. He took no medications.

**Fig. 4:**
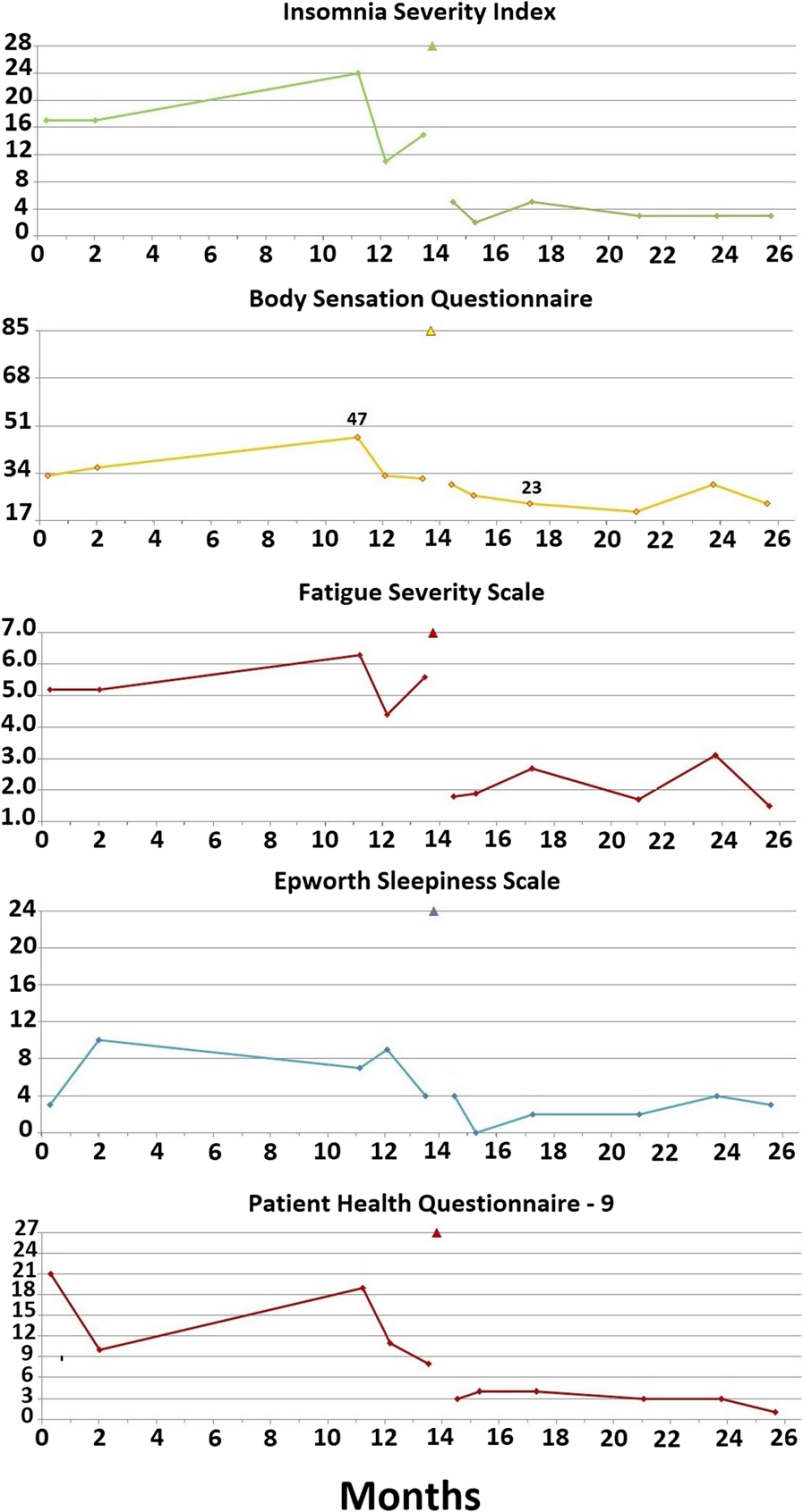
Scores of the same four questionnaires illustrated in Fig. 1: Insomnia Severity Index, Body Sensations Questionnaire, Fatigue Severity Scale, and Epworth Sleepiness Scale, evaluating the severity of insomnia, somatic arousal, fatigue, and sleepiness, respectively. These questionnaires were completed by patient 2 during his evaluation and treatment. The *x*-axis of each graph provides the duration, in months, of our interaction with the patient, and the *y*-axis provides the entire score range for each questionnaire. The date of the surgical procedure is indicated by a marker on the top gridline of each graph. Because patient 2 experienced bipolar disorder, a depression scale, the nine-item Patient Health Questionnaire, is illustrated at the bottom of the figure. The figure indicates a high but somewhat variable level of insomnia severity, somatic arousal, fatigue/sleepiness, and depressed affect for slightly more than 1 year prior to maxillomandibular advancement, with a prompt and persistent decrease in the severity of all symptoms postoperatively. The slight increase in the patient’s stress level (Body Sensations Questionnaire) and fatigue (around month 24) coincides with his relocation to the southwestern United States. The last set of scores was obtained after the relocation was complete

The patient’s ESS score was 3/24, indicating no sleepiness; his FSS score was 5.2/7.0, indicating moderate to severe fatigue; his ISI score of 17/28 characterized severe sleep onset insomnia without sleep maintenance problems; and his BSQ score of 33/85 was consistent with the presence of somatic arousal. The patient completed a self-report depression scale, the Patient Health Questionnaire-9 (PHQ-9), on which he scored 21/27, severe depression, with suicidal ideation more than half his days. Therefore, the questionnaire data reflected severe depression with severe sleep onset insomnia, characterized by somatic arousal and severe fatigue (Fig. 4).

On physical examination, the patient’s height was 178 cm, and his weight was 109 kg (BMI 34.4 kg/m^2^). The patient had gained 18 kg over the preceding 6 months after severe back pain limited his activity. His blood pressure was 127/80 mmHg with a peripheral pulse of 80. His oral airway was characterized by a high-arched, mildly narrowed, hard palate with an elongated soft palate and a Mallampati score of 3. The remainder of his physical examination was unremarkable.

On the morning following the patient’s polysomnogram, he claimed that it had taken him a “long time” to fall asleep and that he had slept, lightly, for only 3 to 4 hours. The polysomnogram demonstrated a sleep latency of 28 minutes (the upper limit of normal is 30 minutes), a total sleep time of 375 minutes, and a sleep efficiency of 89% (normal). N2 sleep percentage was slightly increased (60%); REM latency was prolonged (time to REM sleep onset 148.5 minutes); and the percentage of REM sleep was decreased (14%). The patient’s apnea-hypopnea index was 6.2 with a RDI of 12.2, consistent with mild OSA [20].

To treat the patient’s mild OSA, we prescribed nasal autotitrating CPAP (APAP) with pressure limits of 4 cmH_2_O and 8 cmH_2_O. Simultaneously, he was referred to a maxillofacial surgeon to be considered for curative MMA. After weeks of attempting to sleep with nasal APAP, the patient was unable. We referred the patient to a dentist for a mandibular advancement device, and he went for a consultation, but he did not purchase a device. The maxillofacial surgeon told the patient that he was a candidate for MMA but that he would first have to have his wisdom teeth removed and allow 6 months for healing. He elected to have his wisdom teeth removed.

While the patient waited for 6 months, his symptom pattern continued unabated. He experienced several consecutive days “without sleep” followed by a better night but without an improvement in his daytime fatigue or his affect. He worried that his intellectual capacity was deteriorating. He also expressed skepticism about his insomnia improving after MMA.

Approximately 1 year after first being seen, the patient returned to the SBUSDC. His symptoms were always worse during the late fall and winter, and he sensed they were worsening. He complained of severe insomnia, fatigue, and sleepiness with tension headaches every other day and chronic back pain that was being treated with injections. His ISI score was 24/28, and his BSQ score was 47/85, indicating severe insomnia with marked somatic arousal. His FSS score was 6.3/7.0, indicating severe fatigue, and his PHQ-9 score was 17/27, indicating moderately symptomatic depression (Fig. 4). The patient was encouraged to proceed with MMA as a curative procedure for all his symptoms, and he left the appointment committed to proceeding with MMA.

**Fig. 5:**
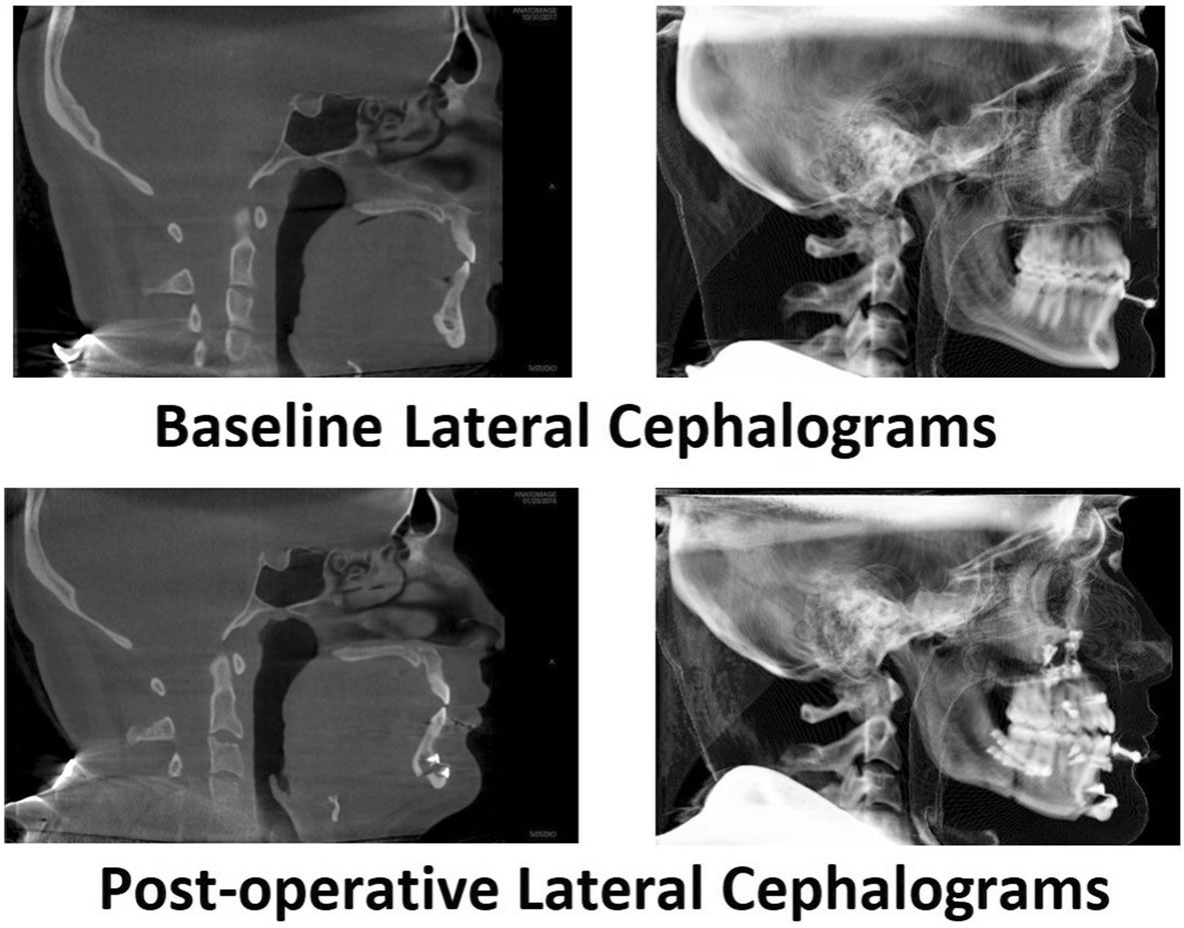
The lateral cephalograms of patient 2 before and after maxillomandibular advancement. The two cephalograms on the left are midsagittal views highlighting the soft-tissue structures. At baseline, the patient demonstrated narrowing of airway at the base of the tongue, with widening after the procedure. The two cephalograms on the right highlight the bony anatomy and illustrate the LeFort 1 procedure, the bilateral sagittal split ramus osteotomy of the mandible, and the genioplasty (*see* Fig. 3 legend)

The patient’s MMA was performed 13 months following his initial sleep consultation. The procedure included a LeFort 1 maxillary advancement, a bilateral sagittal split ramus osteotomy to elongate the body of the mandible, positioning the tongue forward, and an anterior inferior mandibular osteotomy (genioplasty) to further advance the tongue and create a more prominent chin (the procedure illustrated in Fig. 3). Figure 5 demonstrates the patient’s upper airway anatomy before and after surgery.

The effect of MMA on the patient’s insomnia came quickly. At 6 weeks postoperatively, the patient observed “more restful sleep,” feeling tired and ready for bed in the late afternoon/evenings and awakening more easily in the morning. Preoperatively, he had required a very loud alarm to arouse him, whereas postoperatively, he awakened to his vibrating cellular phone. Overall, the patient felt better, and his family noticed. At 7 months postoperatively, the patient slept an average of 8 hours per night, which he considered a “fantastic” outcome.

An unanticipated effect of the MMA procedure was a large weight loss. The patient observed that although he did not diet or increase his exercise, he was not “constantly craving calories,” and he ate “significantly less,” going longer between snacks and meals. The patient lost 19 kg between his initial visit and his final visit 23 months later.

By 7 months, the patient’s affect had greatly improved, and his confidence in his improved health had greatly increased. He was accepted into an online college program and made plans to relocate from Long Island to a city in the southwestern United States, where his opportunities to balance work with school and recreation were better. The patient’s questionnaire scores over the year following surgery (Fig. 4) demonstrate the rapid and persistent resolution of his insomnia, fatigue, somatic arousal (BSQ), and depression.

Polysomnography was performed 9 months postoperatively. On the morning following the study, the patient claimed not to know how long it took to fall asleep and that he slept poorly compared with home, for 5 to 6 hours. The polysomnogram demonstrated a sleep latency of 7.5 minutes, a total sleep time of 346.5 minutes, and a sleep efficiency of 75%. Compared with his first polysomnogram, the patient’s sleep was less consolidated. After initially falling asleep for 45 minutes, the patient awakened for nearly 2 hours before returning to sleep for the rest of the night. N2 sleep percentage was slightly increased (58%); REM latency was prolonged (223.5 minutes); and the percentage of REM sleep was normal (19%). The patient continued to have sleep-disordered breathing with an apnea-hypopnea index of 3.3 and an RDI of 6.8, no longer meeting criteria for OSA (RDI ≥ 15 in an asymptomatic individual) [20]. The patient’s worsened sleep maintenance was attributed to a “first-night effect,” the lighter, less continuous sleep of a “healthy” individual sleeping in a novel environment [21, 22].

### Patient 3

Patient 3 was a 27-year-old Caucasian woman referred for a sleep evaluation by her maxillofacial surgeon, to whom she was referred for temporomandibular joint (TMJ) arthroplasty after years of bruxism. At age 15 years, she had orthodontic work performed and was told that she needed “TMJ surgery,” but she did not have a procedure at that time.

Upon sleep consultation, the patient acknowledged being a lifelong “poor sleeper.” She went to bed at 9:30 p.m. after taking a magnesium supplement and fell asleep in 30 to 60 minutes. Around her menstrual period, however, her sleep latency increased greatly, and she claimed to have episodes of complete insomnia lasting from 3 days to 1.5 weeks. While awaiting sleep, the patient acknowledged discomfort in her legs and episodically experienced visual hypnagogic hallucinations coupled with paralysis. Once asleep, the patient was restless, snoring, grinding her teeth, and experiencing apnea, which occasionally aroused her in a breathless state. Approximately three times weekly, she experienced a recurring nightmare of being trapped/harmed. The patient also had sleep maintenance insomnia, awakening three or four times per night and taking one-half hour, each time, to return to sleep.

The patient awakened at 7:00 a.m. unrefreshed, despite usually having slept 8 hours. She awakened with nasal congestion, a dry mouth, and sore throat and, often, a headache. During the day, she was both sleepy and fatigued, particularly following nights when she did not sleep at all. When driving, she could fall asleep at red lights and experienced periods when she believed she was awake but had no recall of events.

The patient had a deviated nasal septum with rhinitis/sinusitis and seasonal nasal allergies. She also experienced anxiety and was prescribed fluoxetine (20 mg) by her gynecologist, her only medication, which provided some relief.

**Fig. 6:**
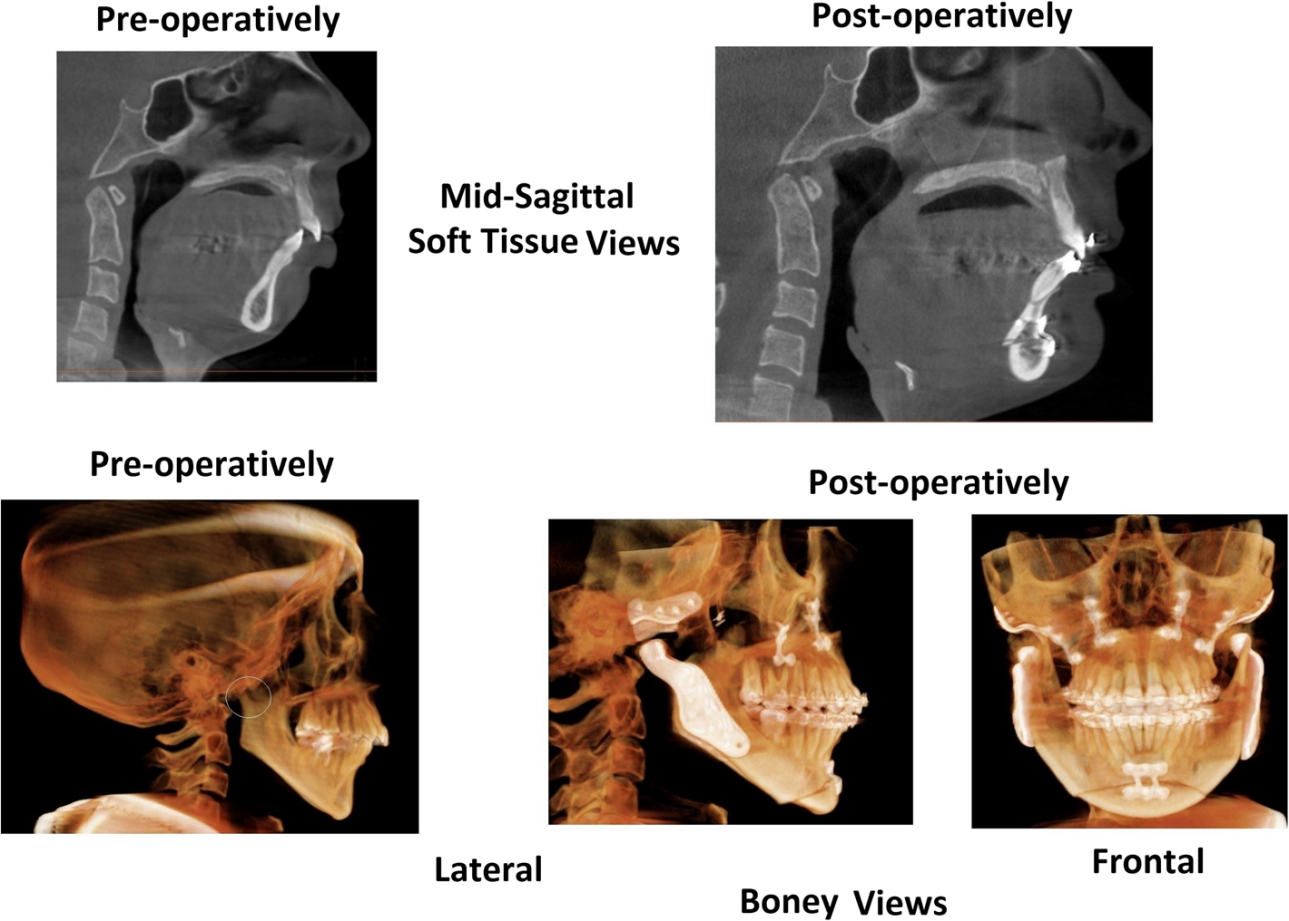
Patient 3’s oropharyngeal anatomy before and after her maxillofacial surgery to replace her temporomandibular joints and relieve her obstructive sleep apnea. Preoperatively, the patient’s degenerating mandibular condyles can be seen disarticulated from the glenoid fossa of the temporal bone, circled with a fine white line in the lateral boney view. Consequently, the mandibular body (and the chin, in the lateral soft tissue view) has receded, and the airway is markedly narrowed. The surgical procedure included an arthroplasty, a LeFort 1 osteotomy, and an anterior inferior mandibular osteotomy (genioplasty; *see also* Fig. 5). The custom-made condylar prostheses served both to articulate with the glenoid fossa and to advance the mandibular body, making a bilateral sagittal split ramus osteotomy unnecessary (*see* Fig. 3 legend). The lateral and frontal boney views show the hardware implanted during the surgery and the braces applied by the orthodontist. The postoperative soft-tissue view shows the elongation of the jaw line and the widened pharyngeal airway

On physical examination, the patient was 158 cm in height and weighed 59 kg (BMI 23.6 kg/m^2^). Her blood pressure was 111/69 mmHg, and her peripheral pulse was 70. Her upper airway was remarkable for disarticulated TMJs with a posteriorly displaced mandible (Fig. 6; preoperative views). Her oral airway was characterized by a Mallampati score of 3 with a narrow, high-arched hard palate and 1+ tonsils.

**Fig. 7:**
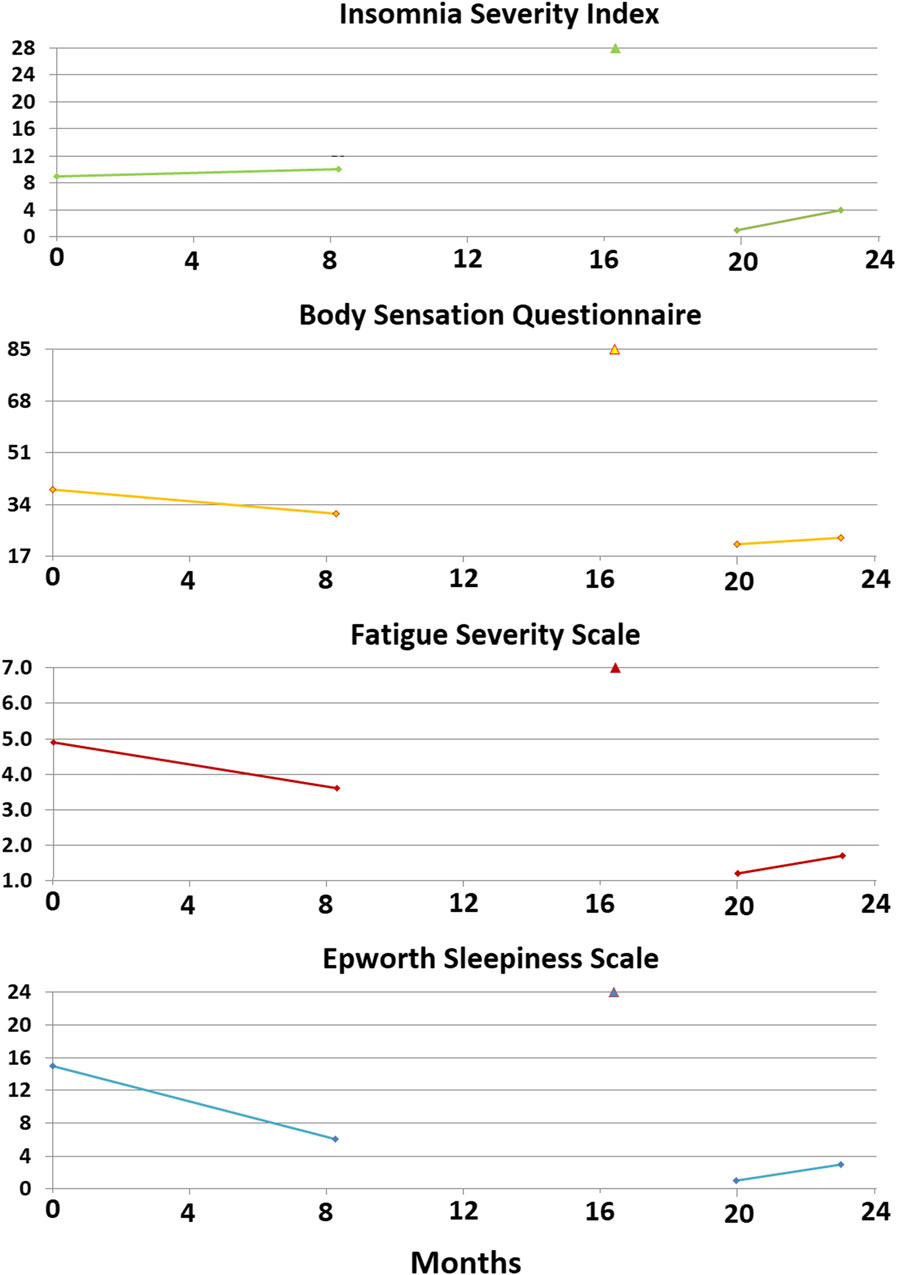
Patient 3’s scores on the same four questionnaires completed by patients 1 and 2 over the course of her treatment (*see* Fig. 1 legend). After the baseline set of questionnaires, the patient was placed on nasal autotitrating continuous positive airway pressure, which she used, with outstanding compliance, for 6 months before completing the second set of questionnaires. Postoperatively, all the patient’s scores improved to minimally symptomatic levels. The date of the surgical procedure is indicated by a marker on the top gridline of each graph

The patient’s questionnaire evaluation revealed an ESS score of 15/24, or moderate sleepiness; an FSS score of 4.9/7.0, or moderate fatigue; an ISI score of 9/28, subclinical insomnia (the patient’s visit did not coincide with the severe insomnia at the time of her period); and a BSQ score of 39/85, increased somatic arousal (Fig. 7).

Because of the patient’s complaints suggesting sleep-disordered breathing, restless legs syndrome, and hypnagogic hallucinations with paralysis (symptoms of narcolepsy), the patient underwent diagnostic polysomnography followed by a multiple sleep latency test (MSLT). Her fluoxetine was not discontinued before the studies.

On the night of the polysomnogram, the patient spent 499 minutes in bed sleeping for 364.5 minutes, a sleep efficiency of 73%. Her sleep latency was prolonged at 102 minutes. NREM sleep was characterized by a decreased percentage of N2 (36%) and increased N3 (33%). REM latency was increased at 132.5 minutes with a normal percentage of REM sleep (24%). The patient snored with an apnea hypopnea index of 5.8 and an RDI of 10.9. No leg movements consistent with periodic leg movement disorder were observed. During arousals from sleep, the electromyographic (EMG) activity of the scalp was markedly increased, and there were episodic bursts of increased chin EMG activity throughout the study, but the short, repetitive bursts of chin EMG activity commonly observed with bruxism were absent. During the MSLT, the patient did not sleep during four of five nap attempts. The sleep latency of the remaining nap was 17 minutes with no REM onset. The patient was diagnosed with mild OSA associated with chronic insomnia and TMJ syndrome.

Because the patient’s OSA may have predated the destruction of her TMJs (her bruxism may have been an indicator of pharyngeal obstruction during sleep), the maxillofacial surgeon decided to perform a LeFort 1 osteotomy with maxillary advancement and genioplasty along with bilateral TMJ arthroplasty. The arthroplasty was done using custom-made condylar prostheses that not only replaced the patient’s mandibular condyles but also pushed the mandibular body forward, making a bilateral sagittal split ramus osteotomy unnecessary. Before surgery, the maxillofacial surgeon referred the patient for corrective orthodontics to align her postoperative bite (Fig. 6). While awaiting surgery, the patient was prescribed nasal APAP with pressure limits of 5 cmH_2_O and 8 cmH_2_O, an appropriate pressure range for her mild OSA.

The patient returned for follow-up approximately 6 months after beginning nasal APAP. Her compliance report for the 30 days before her appointment demonstrated daily use of APAP with nasal pillows averaging 8 hours and 42 minutes per night. Her median nasal pressure was 6.6 cmH_2_O with 95% of the night spent at pressures below 7.9 cmH_2_O (her upper pressure limit was 8.0 cmH_2_O). The patient reported that her usual sleep latency had decreased to 10 minutes since starting nasal APAP, and she no longer experienced hypnagogic hallucinations with paralysis. During her menses, she no longer stayed awake “for days” but still took “2 to 3 hours” to fall asleep. During sleep, she no longer awakened choking and gasping, but she believed she was still bruxing. Her nocturnal awakenings were slightly decreased in frequency, and she was able to return to sleep within 10 minutes. In the morning, she no longer awakened with nasal congestion and a headache. Overall, she believed her ability to focus and concentrate had improved, but her anxiety had not improved.

The patient’s questionnaire scores reflected her improved condition (Fig. 7). Her ESS score had decreased to 6/24. Her FSS score had decreased to 3.6/7.0, and her BSQ score had decreased to 31/85. Her ISI score was unchanged at 10/28. The persistently elevated level of somatic arousal and fatigue were consistent with the patient’s continued, if milder, insomnia and anxiety.

Approximately 8 months following her follow-up visit, the patient’s arthroplasty/MMA was performed. The postoperative views in Fig. 6 demonstrate the surgical and orthodontic work performed. Immediately postoperatively, the patient discontinued her nasal APAP, and 6 weeks postoperatively, she had repeat polysomnography.

On the night of the postoperative polysomnogram, the patient spent 477.5 minutes in bed, sleeping for 422.5 minutes, a normal sleep efficiency of 88%. Her sleep latency was greatly decreased from her initial study to 21.5 minutes. Compared with her initial polysomnogram, her NREM sleep became lighter with increased N1 (26%), normal N2 (55%), and decreased N3 (5%). REM latency remained prolonged at 177 minutes with decreased REM sleep (14%). The patient’s apnea-hypopnea index decreased to 0.9 with an RDI of 3.0, below the threshold for a diagnosis of OSA. The patient’s lighter sleep with delayed, decreased REM sleep was attributed to a “first-night effect” occurring in a “healthy” patient sleeping for the first time in a novel environment [21, 22].

The patient returned to the SBUSDC approximately 2 months following her postoperative polysomnogram. She reported further improvement in her symptoms from their levels using nasal APAP, complete resolution of her insomnia (even during her menses), and further improvement in her cognitive ability and anxiety (although she had not discontinued fluoxetine). Her questionnaire scores reflected her marked improvement, with the ESS score, FSS score, BSQ score, and ISI score all minimized. The improvement was reconfirmed by another set of questionnaire scores 3 months later (Fig. 7).

## Discussion and conclusions

In this report of three patients, we have presented cases of the co-occurrence of chronic insomnia and OSA. Two patients presented specifically for their severe, chronic insomnia, with neither acknowledging snoring or witnessed apnea, whereas the third was referred by her surgeon before TMJ arthroplasty to address possible underlying OSA. All patients were treated specifically for their mild OSA with no intervention directed at their chronic insomnia. When their OSA was effectively treated by maxillofacial surgery, all experienced prompt reduction of their somatic arousal and relief of their chronic insomnia. The clinical experiences of these three patients are consistent with a paradigm of OSA as a disorder of chronic stress [12] characterized by somatic arousal that may manifest as chronic insomnia in those with both disorders [15].

**Fig. 8:**
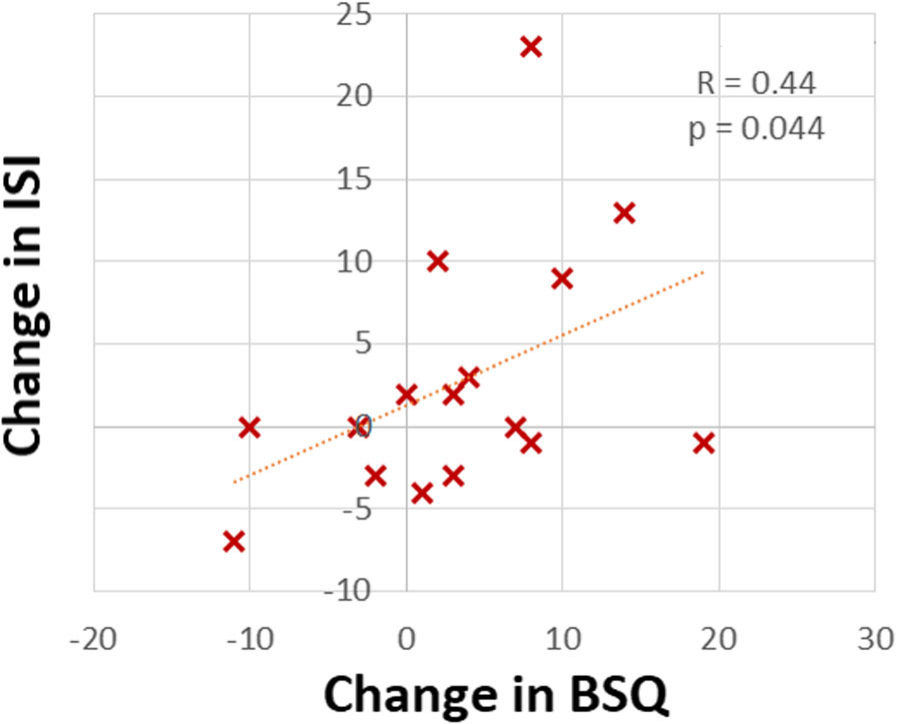
The changes in Body Sensations Questionnaire scores (BSQ; change in somatic arousal) versus the changes in Insomnia Severity Index scores (ISI) for our three patients over the course of their treatment and follow-up. To determine each data point, a pair of simultaneously obtained BSQ and ISI scores was subtracted from the immediately preceding pair of simultaneously obtained BSQ and ISI scores. The 16 values plotted are evident in Figs. 1, 4, and 7, with patients 1 and 3 each supplying 3 data points and patient 2 supplying 10 data points. The figure demonstrates a statistically significant positive correlation between the changes in somatic arousal level experienced by our patients and the changes in severity of their chronic insomnia

That somatic arousal has a causative role in chronic insomnia is a long-held paradigm of behavioral sleep medicine [1]. Insomnia investigators have identified many markers for somatic arousal that are increased in patients with chronic insomnia compared with control subjects. Among these markers are increased heart rate [23] and decreased heart rate variability [24], markers for increased SNS activity. Although investigators have identified increased somatic arousal among patients with chronic insomnia, how the level of somatic arousal relates to insomnia severity is unclear. In this case series, we have measured somatic arousal repeatedly with the BSQ and simultaneously measured insomnia severity with the ISI (Figs. 1, 4, and 7). Therefore, we can correlate changes in the level of somatic arousal with changes in the severity of chronic insomnia in our three patients. Figure 8 plots the changes in our patients’ BSQ scores with changes in their simultaneously measured ISI scores and demonstrates a significant positive correlation between the two parameters (*R* = 0.44; *P* = 0.044). Although not addressing cause and effect (does the stress level, measured as somatic arousal, determine the severity of insomnia, or does the severity of insomnia determine the stress level?), the correlation is consistent with a role for the level of somatic arousal determining the severity of insomnia among patients with both OSA and chronic insomnia.

Before concluding, we will address a unique feature of this three-patient series: the treatment of mild OSA with MMA. Indeed, the medical literature is uncertain about whether to treat mild OSA at all [25, 26]. It is evident in these case reports that we are very comfortable treating young, highly symptomatic patients with mild OSA surgically to ameliorate their upper airway obstruction and preclude a lifetime of nasal CPAP. Our comfort comes from our recognition that OSA is a disorder of chronic biological stress with inspiratory airflow limitation during sleep as the stressor [12]. We are unconcerned by the OSA severity (mild, moderate, severe) based on the classical sleep fragmentation paradigm of OSA. Rather, we are focused on the level of chronic stress manifesting as somatic arousal and measured by the BSQ score. We believe that patients with mild OSA who present with high levels of chronic stress accompanied by sleep onset and maintenance insomnia [15] and fatigue [14] are candidates for surgical intervention to ameliorate the upper airway obstruction and relieve the symptoms, as observed in our patients.

## Data Availability

All the data in this case series are maintained in HIPAA-protected locations except for the email correspondence between patient 2 and the sleep medicine physician (ARG), which is archived (with backup) on the physician's computer at the Northport DVA Medical Center, Northport, NY 11768 (federally protected). Every piece of clinical data obtained by Stony Brook University (SBU) Sleep Disorders Center, including all questionnaire scores, exist in the SBU Medical Center electronic medical record. The radiographs used for the figures reside in the patient records at Stony Brook Oral and Facial Surgery.

## Abbreviations

AIMO: Anterior inferior mandibular osteotomy
APAP: Autotitrating continuous positive airway pressure
BMI: Body mass index
BSQ: Body Sensations Questionnaire
CPAP: Continuous positive airway pressure
EMG: Electromyographic
ESS: Epworth Sleepiness Scale
FSS: Fatigue Severity Scale
ISI: Insomnia Severity Index
LF: LeFort 1
MMA: Maxillomandibular advancement
MSLT: Multiple sleep latency test
NREM: Non-rapid eye movement
OSA: Obstructive sleep apnea
PHQ-9: Patient Health Questionnaire-9
RDI: Respiratory disturbance index
REM: Rapid eye movement
SBUSDC: Stony Brook University Sleep Disorders Center
SNS: Sympathetic nervous system
TMJ: Temporomandibular joint
